# Living conditions and autonomy levels in COPD patients receiving non-invasive ventilation: impact on health related quality of life

**DOI:** 10.1186/s12890-021-01621-4

**Published:** 2021-08-03

**Authors:** Sarah Bettina Schwarz, Tim Mathes, Daniel Sebastian Majorski, Maximilian Wollsching-Strobel, Doreen Kroppen, Friederike Sophie Magnet, Wolfram Windisch

**Affiliations:** 1 Department of Pneumology, Cologne Merheim Hospital, Kliniken der Stadt Köln gGmbH, Witten/Herdecke University, Faculty of Health/School of Medicine , Ostmerheimer Strasse 200, 51109 Cologne, Germany; 2 Institute for Research in Operative Medicine, Faculty of Health - School of Medicine, Witten/Herdecke University, Ostmerheimer Strasse 200, 51109 Cologne, Germany

**Keywords:** Non-invasive ventilation, Quality of life, Impairment of autonomy, COPD

## Abstract

**Background:**

Research on health-related quality of life (HRQL) has become increasingly important in recent decades. However, the impact of both living conditions and the level of autonomy impairments on HRQL in COPD patients receiving non-invasive ventilation (NIV) is still unclear.

**Methods:**

The Severe Respiratory Insufficiency Questionnaire (SRI) was used to measure HRQL in a prospective cohort of COPD patients in whom home NIV was already established. Data on sociodemographics, clinical characteristics and standardized levels of autonomy impairment were evaluated. A multiple linear regression analysis was performed to identify the factors associated with a reduced HRQL.

**Results:**

A total of 137 patients (67.0 ± 7.8 years, 45% female) were assessed. The mean SRI Summary Score was 54.1 ± 16.9 (95%CI: 51.1–57.1; N = 127). Regular ambulatory care was provided in 76% of patients, but only 37% underwent pulmonary rehabilitation. Overall, 69% of patients lived with family members, while 31% lived alone (family situation). Autonomy impairment levels were most serious in 3%, serious in 14%, and significant in 29% of patients, while 54% had no impairments at all. Of note, higher levels of autonomy impairment were markedly associated with lower SRI scores (regression coefficient − 6.5 ± 1.1 per level; *P* < 0.001). In contrast, family situation (0.2 ± 3.0; *P* = 0.959), ambulatory care by a respiratory specialist (1.7 ± 3.6; *P* = 0.638), and pulmonary rehabilitation (− 0.8 ± 3.1; *P* = 0.802) did not appear to influence HRQL. Possible subgroup effects were evident for the factors “impaired autonomy” and “living in a nursing home” (*P* = 0.016).

**Conclusion:**

A higher level of autonomy impairment has been identified as the major determinant of reduced HRQL in COPD-patients receiving long-term NIV, particularly in those living in a nursing home.

*Trial Registration* German Clinical Trials Register (DRKS00008759).

**Supplementary Information:**

The online version contains supplementary material available at 10.1186/s12890-021-01621-4.

## Background

Long-term non-invasive ventilation (NIV) serves as an established treatment option for patients with chronic respiratory failure arising from various etiologies [1, 2]. COPD patients represent the largest proportion of those receiving long-term NIV therapy [3]. These patients are typically characterized by several co-morbidities, advanced age and severely reduced health-related quality of life (HRQL) [4–7]. Nevertheless, recent evidence suggests that long-term NIV is beneficial for these patients because it demonstrates the potential of long-term NIV to improve HRQL. [8, 9]. These positive therapy effects on HRQL are particularly evident in COPD patients [10]. However, a recently published meta-analysis reveals inconsistent data, particularly regarding quality of life outcomes due to a lack of disease-related studies [11].

Evidence for the potential benefits of long-term NIV therapy in chronic hypercapnic COPD patients is primarily derived from randomized controlled trials (RCTs) [8]. However, the number of patients ultimately randomized into these studies is typically lower than the number initially screened. For example, according to the landmark study by Murphy and colleagues on hospital readmission or death after an acute COPD exacerbation episode, 296 patients refused to participate, while only 116 patients were eventually randomized. In addition, many other patients (N = 1609) did not end up being randomized for a number of other reasons [12]. Thus, the process for allocating patients to RCTs is generally highly selective, and this also likely pertains to studies investigating complex interventions such as long-term NIV in severely ill patients who usually have a reduced level of autonomy. More specifically, it is possible that patients with impaired autonomy are less likely to be included in RCTs designed to assess the impact of long-term NIV in COPD patients [13].

Furthermore, it has been reported that COPD patients with both the highest degree of autonomy impairment and the need for long-term invasive mechanical ventilation following weaning failure have a severely compromised HRQL [14, 15]. Thus, a lower level of autonomy could have a negative impact on HRQL in COPD patients undergoing long-term NIV. This, however, has not yet been systematically investigated. In addition, it is unclear if HRQL is well preserved in NIV-dependent COPD patients who: (i) live in a nursing home, (ii) live alone instead of with family members, and (iii) do not receive regular outpatient care.

The aim of the present study was to evaluate the determinants of HRQL in patients with COPD on long-term NIV and to assess the health care infrastructure. It was hypothesized that long-term NIV-related HRQL is lower if: (i) patients do not receive regular outpatient care from a respiratory specialist, (ii) patients have not undergone pulmonary rehabilitation, (iii) patients live alone, and (iv) the patient's degree of autonomy is significantly impaired. If so, further RTCs would be needed to warrant the use of long-term NIV as a means of improving HRQL in patients with restricted autonomy and/or unfavourable living conditions.

## Methods

This prospective, single-centre, observational cohort study was conducted at the Department of Pneumology at Cologne-Merheim Hospital, University Witten/Herdecke. The study protocol was approved by the Institutional Review Board for Human Studies at the University of Witten/Herdecke, and was performed in accordance with the ethical standards laid down in the Declaration of Helsinki (last revision: 2013) [16]. Written informed consent was obtained from all subjects or their legal guardian. The study was prospectively registered at the German Clinical Trials Register (DRKS00008759). The results presented in this article are reported according to the STROBE Statements for cohort studies [17]. The corresponding STROBE statement is available in the Additional file 1: Table S1.

**Table 1:** Patient characteristics and NIV data

(n = 137)	
No. of females (%)	62 (45%)
Age (years)	67.0 ± 7.8
Body-mass index (kg/m^2^)	27.9 ± 8.2
Smoking status (n; active:prior)	29:108
(%; active:prior)	21.2:78.8
Smoking index (Pack Years)	56.3 ± 24.9
Time under NIV (years)	2.1 ± 2.6
Supplemental Oxygen (n; yes:no)	117: 22
(%; yes:no)	84.2:15.8
LTOT (l/min during rest; n = 117)	2.1 ± 0.8
NIV initiation	
Chronic elective NIV	59 (43%)
Following acute NIV	73 (53%)
NIV following prolonged weaning	5 (4%)
Ventilator settings	
IPAP (cmH_2_O)	23.6 ± 4.6
EPAP (cmH_2_O)	5.9 ± 1.3
BF (per minute)	16.5 ± 2.4
Adherence (mean hours per day)	6.5 ± 3.1
Compliance* (n; compliant: not compliant)	109:28
(%; compliant, not compliant)	79.6:20.4

No.: Number; LTOT: long-term oxygen therapy; IPAP: inspiratory positive airway pressure; EPAP: expiratory positive airway pressure; BF: breathing frequency; NIV: non-invasive ventilation; * Patients were defined as non-compliant if NIV use was less than 4 h/day

### Subjects

The data were collected over a period of five years from June 2015 to July 2020. Patients who had already been established on non-invasive home mechanical ventilation according to national [1, 2] and international guidelines and recommendations [8] were consecutively screened for eligibility during outpatient follow-up visits or inpatient admissions. Only clinically stable patients in whom COPD was the primary underlying disease that led to the need for long-term NIV were included in the study. Non-COPD patients as well as those with an acute exacerbation (according to GOLD Guidelines) were excluded from the study [18].

### Sample size and data collection

Since the study was planned as a pilot study, no formal sample size calculation was performed. At least 120 patients were planned for inclusion to ensure a reliable basis for the statistical analysis of a further study [19].

Data on sex, age, duration of NIV, and the condition associated with NIV initiation were collected for each of the following three groups: (1) patients with stable chronic hypercapnia, (2) patients with persistent hypercapnia following acute hypercapnic exacerbation, and (3) patients with prolonged and unsuccessful weaning; smoking status and index, long-term oxygen therapy (LTOT), ventilator settings and daily use of NIV. Patient adherence defined as the mean time in hours of use per day was determined by a readout of the ventilator’s built-in software. Oxygen was supplied both according to the patient’s individual needs and based on national and international guidelines [20–22].

HRQL was assessed by the Severe Respiratory Insufficiency Questionnaire (SRI), which was specifically developed and validated for patients with long-term NIV [6, 23]. The SRI has been shown to be particularly valuable for patients with COPD and chronic respiratory failure who require treatment with LTOT and/or long-term NIV [6, 24]. The SRI contains 49 items with 7 subscales that measure different aspects of HRQL (Respiratory Complaints, Physical Functioning, Attendant Symptoms and Sleep, Social Relationships, Anxiety, Psychological Well-Being, Social Functioning). Each subscale produces a score (0–100), with lower scores indicating a poorer HRQL. The subscales can be aggregated into one Summary Scale.

Following topic-focused interviews, further information on both home environment (nursing home vs. private home) and family situation (living alone vs. living with any family member) was provided; occupational status was also assessed. Finally, the use of the different parts of the health care infrastructure was determined: 1. Had patients ever received pulmonary rehabilitation? (yes vs. no), and 2. Does regular ambulatory care by a respiratory specialist take place? (yes vs. no).

Finally, the level of autonomy impairment was graded for each patient. For this purpose, the German health care system requires that all patients with impairments are externally evaluated by the Health Insurance Medical Service (Medizinischer Dienst der Krankenversicherung) according to standard criteria [25]. Accordingly, five impairment levels are defined: minor impairments (level 1), significant impairments (level 2), serious impairments (level 3), most serious impairments (level 4), most severe impairments to independence or ability that are associated with significant challenges for nursing care (level 5).

### Data management and statistical analysis

Once collected, all data were entered into a standardised case report form (CRF), subsequently documented in a pseudonymous fashion, and archived electronically. The impact of the patient's care, family situation, and level of autonomy on HRQL was assessed. SRI scores were used only for the calculation if the proportion of missing values were less than 10%.

For the purpose of the study, a descriptive univariate analysis was performed by calculating the mean for each group classified by the manifestation of the variable separately (e.g. respiratory specialist, yes vs. no), with their 95% confidence interval (95% CIs). A multiple linear regression analysis was subsequently performed to assess the adjusted impact of the individual variables on HRQL. As a supplemental analysis, a linear regression analysis was conducted to investigate possible effects of different combinations of health care indicators.

## Results

**Fig. 1:**
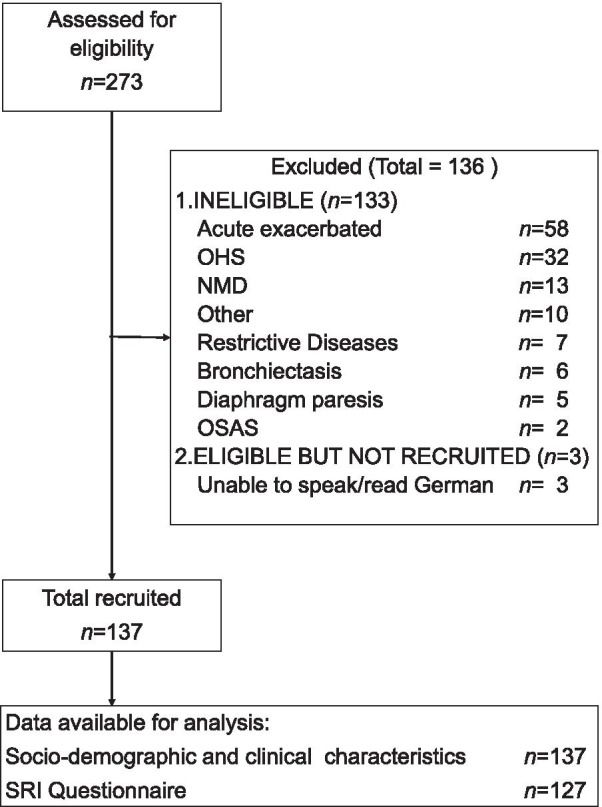
Flow diagram of subject recruitment and data availability. n Number; OSAS obstructive sleep apnea syndrome; OHS obesity hypoventilation syndrome; NMD neuromuscular diseases; SRI severe respiratory insufficiency questionnaire

A total of 273 patients were screened for eligibility, with 136 of them ultimately excluded from the study (Fig. 1). A total of 137 patients with COPD and pre-existing NIV were enrolled, with 127 patients (91%) having completed the SRI. Complete data sets were analysed to determine the attributes “level of autonomy impairment”, “pulmonary rehabilitation” and “family situation”. For the attribute “ambulatory care by a respiratory specialist”, data from one patient was not entered (data available from the remaining 99.3%).

**Fig. 2:**
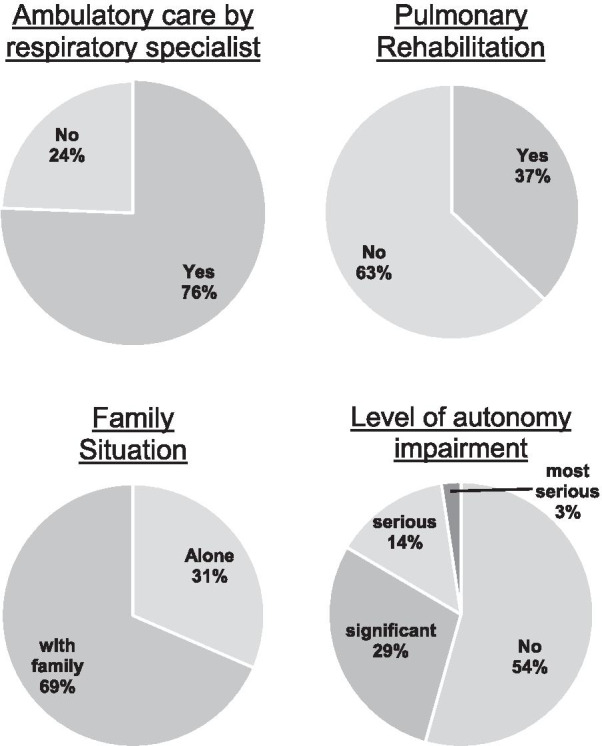
Patient care, family situation and autonomy level in COPD patients receiving long-term NIV (*N* = 137)

Patient characteristics and data on NIV treatment are presented in Table 1. Regarding occupational status, 10% of patients were still employed or employable, while 24% were unemployable and 66% were retired. In addition, 95% of the patients lived in a private home environment, while just 5% lived in a nursing home. Data on patient care, family situation and autonomy level are illustrated in Fig. 2. Of note, 69 patients (54%) did not qualify for any level of impairment as externally graded by the Health Insurance Medical Service. Conversely, 58 patients (46%) were assigned an externally defined level of impairment, as outlined in Fig. 2. The mean SRI score was 54.1 ± 16.9 (95%CI 51.1–57.1). Detailed data on HRQL are provided in Table 2.

**Table 2:** Severe Respiratory Insufficiency Questionnaire (SRI) scores (*n* = 127)

	Mean	SD	95% CI	
			Lower limit	Upper limit
Respiratory complaints	53.8	19.9	50.3	57.3
Physical functioning	37.1	24.2	32.9	41.4
Attendant symptoms and sleep	58.4	19.1	55.0	61.7
Social relationships	68.6	21.7	64.8	72.5
Anxiety	51.1	24.1	46.9	55.4
Psychological well-being	57.7	19.3	54.3	61.1
Social functioning	50.8	22.4	46.9	54.8
Summary scale	54.1	16.9	51.1	57.1

CI Confidence interval; SD standard deviation

**Fig. 3:**
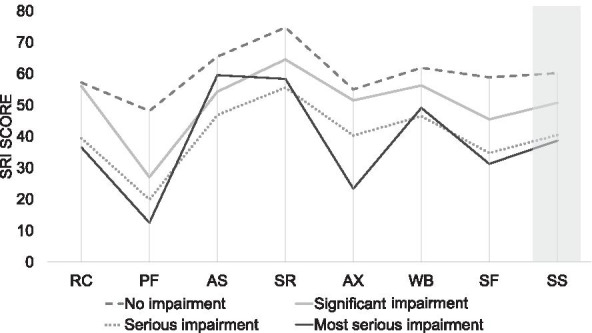
Domains of the Severe Respiratory Insufficiency (SRI) Questionnaire in patients receiving long-term non-invasive ventilation in the home setting (n = 127). RC: Respiratory complaints; PF: physical functioning; AS: attendant symptoms and sleep; SR: social relationships; AX: anxiety; WB: psychological well-being; SF: social functioning; SS: summary scale

The results of univariate analysis of the SRI Summary Scale in terms of patient care, family situation and autonomy level are presented in Table 3. A multiple linear regression analysis of the SRI Summary Scale in relation to patient care, family situation and autonomy level is presented in Table 4. Importantly, SRI Summary Scale scores were mainly influenced by the level of autonomy impairment. Here, a higher level of impairment was predictive for a lower SRI Summary Scale score (− 6.5 ± 1.1; *p* ≤ 0.001), whereas family situation (0.2 ± 3.0; *p* = 0.959), no routine visits by pneumologist (1.7 ± 3.6; *p* = 0.638) or pneumological rehabilitation (− 0.8 ± 3.1; *p* = 0.802) was not associated with lower HRQL. The results of the SRI subscale scores are shown in Additional file 1: Tables S2, S3 and S4. The strong reductions in HRQL in patients with a higher impairment level of autonomy are evident in the subscales for physical functioning and anxiety (Fig. 3). The group of patients living in a nursing home was analysed separately in terms of demographic characteristics (Additional file 1: Table S5). A possible interaction effect was shown, whereby the combination of impaired level of autonomy and living in a nursing home particularly deteriorates HRQL (*p* = 0.016). Likewise, the factors "impairment levels of autonomy" and "living alone" trended towards a possible interaction effect (*p* = 0.097).

**Table 3:** Univariate analysis of the summary score of the severe respiratory insufficiency questionnaire (SRI) in relation to patient care, family situation and autonomy level (*n* = 127)

	Mean SRI	95% CI	
		Lower limit	Upper limit
Ambulatory care by a respiratory specialist			
No	52.4	45.3	59.5
Yes	54.7	51.4	58.0
Rehabilitation			
No	52.3	50.5	58.0
Yes	53.9	48.8	59.0
Family situation			
Alone	53.7	48.5	58.8
With family	54.3	50.6	58.0
Level of autonomy impairment			
No	60.2	56.3	64.0
(1) Minor	–	–	–
(2) Significant	50.7	46.3	55.1
(3) Serious	40.4	32.3	48.6
(4) Most serious	38.6	6.7	70.6
(5) Most severe	–	–	–

CI: Confidence interval

**Table 4:** Multiple linear regression analysis of the summary score of the Severe Respiratory Insufficiency Questionnaire (SRI) in relation to patient care, family situation and autonomy level, adjusted for different variables (n = 127)

		95% CI		
	Regression coefficient	Lower limit	Upper limit	*P* value
Ambulatory care (respiratory specialist)	1.7	− 5.4	8.8	0.638
Age (per additional 10 years)	0.1	− 0.3	0.5	0.607
Family situation (alone vs. with family)	1.2	− 5.4	7.8	0.715
Occupational status*	− 14.8	− 25.0	− 4.6	**0.005**
Home environment (private home vs. nursing home)	4.8	− 8.8	18.4	0.490
Rehabilitation	− 0.8	− 7.0	5.4	0.802
Respiratory specialist (yes vs. no)	2.2	− 4.9	9.3	0.547
Home environment (private home vs. nursing home)	4.4	− 8.7	17.5	0.508
Exacerbations (last 12 months)	− 7.2	− 12.6	− 1.9	**0.008**
Family situation	0.2	− 5.8	6.1	0.959
Level of autonomy impairment^#^	− 8.5	− 11.4	− 5.6	**< 0.001**
Home environment (private home vs. nursing home)	12.2	− 0.3	24.8	0.055
Level of autonomy impairment	− 6.5	− 8.7	− 4.3	**< 0.001**
Home environment (private home vs. nursing home)	13.9	1.2	26.6	**0.032**
Family situation (alone vs. with family)	1.2	− 4.8	7.1	0.693

Significant values are marked in bold

CI: confidence interval; * employed or able to work, retired, unemployable; ^#^ no impairments, significant impairments (level 2), serious impairments (level 3), most serious impairments (level 4)

## Discussion

The results of this study highlight the heterogeneous healthcare landscape of NIV-treated COPD patients, with particular emphasis on HRQL. The three main results are as follows: Firstly, there is lack of patient care following NIV establishment—at least in some patients—since 24% do not have regular outpatient follow-up by a respiratory specialist, while 63% had never undergone pulmonary rehabilitation. Secondly, impairments in autonomy were evident in many patients, with 46% of all patients having significant, serious or most serious impairments, as defined by standard German assessment criteria [25]. Thirdly, and most importantly, when HRQL was specifically measured by the SRI, it was found to be dramatically reduced in patients with higher levels of autonomy impairment, especially in those patients living in a nursing home.

These findings raise some important clinical considerations. The recent task force of the European Respiratory Society on “Long-term home NIV for the management of COPD” suggests the use of long-term NIV in chronic hypercapnic COPD-patients. This is primarily based on scientifically established improvements in subjective measures, which most importantly include HRQL. Several studies that applied the highly specific SRI reported improvements in the SRI Summary Scale after NIV initiation, ranging from no improvements to up to 11 points [12, 26–28]. However, the current study  shows that lower mean SRI scores were associated with higher levels of autonomy impairments. Here, NIV patients with serious and most serious levels of autonomy impairment had lower SRI scores than patients in all previous trials who had hypercapnic COPD prior to NIV commencement [12, 26–29].

Interestingly, a lack of outpatient pulmonary follow-up and rehabilitation did not have an impact on specific aspects of HRQL in COPD patients with long-term NIV. In addition, the patient's family situation did not influence HRQL. Thus, the level of autonomy impairment forms the major determinant of HRQL in these patients. Importantly, both physical and psychological aspects of HRQL were affected by autonomy impairments, particularly in terms of physical functioning and anxiety.

The pertinence of autonomy levels to HRQL has potentially important consequences, whereby we put forward two major suggestions: Firstly, in COPD patients with chronic hypercapnic failure who are subjected to long-term NIV treatment, all efforts should be made to improve the degree of autonomy. However, whether this actually leads to improvements in HRQL should be addressed in future trials. This also holds true for the question of whether pulmonary rehabilitation is capable of ameliorating these impairments and hence HRQL.

Secondly, future studies should address the question of whether long-term NIV therapy also leads to an improvement in HRQL in those patients who continue to have severely impaired levels of autonomy, even after long-term NIV has been established [12, 29]. Especially in patients who continue to have severely impaired levels of autonomy, therapy should be critically evaluated with regard to its necessity and potential modifications of the therapy regimen should also be discussed in collaboration with the patient. Indeed, recent studies have raised ethical concerns about the continuation of long-term mechanical ventilation in COPD patients with long-term invasive mechanical ventilation and severely impaired autonomy, especially when they have a severely reduced HRQL [14, 15, 30, 31]. Of note, these studies also showed that a poor HRQL was associated with the wish to stop mechanical ventilation, which would invariably lead to the death of the patient. Thus, palliative care for those who ultimately waive the option of NIV therapy should also be discussed for this patient group. Overall, this is an important topic that requires further investigation.

One limitation of this study is that it was designed as a single centre investigation in a single country. Even though the generalisation of the findings should be viewed with caution, the impact of autonomy levels on HRQL are suggested to be globally valid, particularly in view of the meaningful and sound results of the present study. In addition, we cannot exclude that the results are influenced by further confounders. Further studies are needed to identify the specific groups of patients who will benefit most from NIV therapy.

## Conclusion

In summary, there are dramatic differences in HRQL amongst COPD patients with established long-term NIV, depending on the degree of autonomy impairment. Patients with either serious or most serious impairments have lower SRI scores than all of those previously reported in RCTs for COPD patients yet to commence NIV therapy. Thus, the present study has clearly identified the association of the level of autonomy impairments and health-related quality of life in severely ill COPD patients undergoing long-term NIV therapy.

## Supplementary Information

**Additional file 1.** Supplementary Tables.

## Data Availability

The datasets used and/or analysed during the current study are available from the corresponding author on reasonable request.

## Abbreviations

CIs: Confidence interval
COPD: Chronic obstructive pulmonary disease
CRF: Case report form
HRQL: Health-related quality of life
LTOT: Long-term oxygen therapy
NIV: Non-invasive ventilation
RCT: Randomized controlled trials
SRI: Severe Respiratory Insufficiency Questionnaire
