# Supplementary material for: Living conditions and autonomy levels in COPD patients receiving non-invasive ventilation: impact on health related quality of life
Source: BMC Pulm Med. 2021 Aug 3;21:255. doi: 10.1186/s12890-021-01621-4 (PMC8330117; doi:10.1186/s12890-021-01621-4)
Supplement: Supplementary file 1 — Additional file 1. Supplementary Tables. [file 12890_2021_1621_MOESM1_ESM.docx]

**Online supplement:**

Table e1: STROBE Statement

|  | **Item No.** | **Recommendation** | **Page  No.** | **Relevant text from manuscript** |
| --- | --- | --- | --- | --- |
| **Title and abstract** | 1 | (*a*) Indicate the study’s design with a commonly used term in the title or the abstract | 1 |  |
|  |  | (*b*) Provide in the abstract an informative and balanced summary of what was done and what was found | 2 |  |
| **Introduction** | | | |  |
| Background/rationale | 2 | Explain the scientific background and rationale for the investigation being reported | 3-4 |  |
| Objectives | 3 | State specific objectives, including any prespecified hypotheses | 4 |  |
| **Methods** | | | |  |
| Study design | 4 | Present key elements of study design early in the paper | 4 | Prospective, single-center, observational cohort study. |
| Setting | 5 | Describe the setting, locations, and relevant dates, including periods of recruitment, exposure, follow-up, and data collection | 4 | The data were collected from June 2015 to July 2020. |
| Participants | 6 | (*a*) *Cohort study*—Give the eligibility criteria, and the sources and methods of selection of participants. Describe methods of follow-up | 4 |  |
|  |  | (*b*) *Cohort study*—For matched studies |  | n/a |
| Variables | 7 | Clearly define all outcomes, exposures, predictors, potential confounders, and effect modifiers. Give diagnostic criteria, if applicable | 5 |  |
| Data sources/ measurement | 8* | For each variable of interest, give sources of data and details of methods of assessment (measurement). Describe comparability of assessment methods if there is more than one group | 5 |  |
| Bias | 9 | Describe any efforts to address potential sources of bias | 5 |  |
| Study size | 10 | Explain how the study size was arrived at | 4 | We planned to include at least 120 patients to ensure a reliable basis for further statistical analysis. |

| Quantitative variables | 11 | | Explain how quantitative variables were handled in the analyses. If applicable, describe which groupings were chosen and why | 5 | | |  |
| --- | --- | --- | --- | --- | --- | --- | --- |
| Statistical methods | 12 | | (*a*) Describe all statistical methods, including those used to control for confounding | 5-6 | | |  |
|  |  |  | (*b*) Describe any methods used to examine subgroups and interactions | 5-6 | | |  |
|  |  |  | (*c*) Explain how missing data were addressed | 6 | | |  |
|  |  |  | (*d*) *Cohort study*—If applicable, explain how loss to follow-up was addressed |  | | | n/a |
|  |  |  | (*e*) Describe any sensitivity analyses |  | | | n/a |
| **Results** | | | | | | | |
| Participants | 13* | | (a) Report numbers of individuals at each stage of study—eg numbers potentially eligible, examined for eligibility, confirmed eligible, included in the study, completing follow-up, and analysed |  | | | Figure 1 |
|  |  |  | (b) Give reasons for non-participation at each stage |  | | | Figure 1 |
|  |  |  | (c) Consider use of a flow diagram |  | | | Figure 1 |
| Descriptive data | 14* | | (a) Give characteristics of study participants (eg demographic, clinical, social) and information on exposures and potential confounders |  | | | Table 1 |
|  |  |  | (b) Indicate number of participants with missing data for each variable of interest | 6 | | |  |
|  |  |  | (c) *Cohort study*—Summarise follow-up time (eg, average and total amount) |  | | | n/a |
| Outcome data | 15* | | *Cohort study*—Report numbers of outcome events or summary measures over time |  | | | n/a |
| Main results | 16 | | (*a*) Give unadjusted estimates and, if applicable, confounder-adjusted estimates and their precision (eg, 95% confidence interval). Make clear which confounders were adjusted for and why they were included |  | | | Table 2-4 |
|  |  |  | (*b*) Report category boundaries when continuous variables were categorized |  | | | n/a |
|  |  |  | (*c*) If relevant, consider translating estimates of relative risk into absolute risk for a meaningful time period |  | | | n/a |
| Other analyses | | 17 | Report other analyses done—eg analyses of subgroups and interactions, and sensitivity analyses | |  | Figures 2 + 3  Tables e2-5 | |
| **Discussion** | | | | | | | |
| Key results | | 18 | Summarise key results with reference to study objectives | | 11-12 |  | |
| Limitations | | 19 | Discuss limitations of the study, taking into account sources of potential bias or imprecision. Discuss both direction and magnitude of any potential bias | | 13 |  | |
| Interpretation | | 20 | Give a cautious overall interpretation of results considering objectives, limitations, multiplicity of analyses, results from similar studies, and other relevant evidence | | 13 |  | |
| Generalisability | | 21 | Discuss the generalisability (external validity) of the study results | | 13 |  | |
| **Other information** | | |  | | | | |
| Funding | | 22 | Give the source of funding and the role of the funders for the present study and, if applicable, for the original study on which the present article is based | | 14 |  | |

No.: number; n/a: not applicable

Table e2: Severe Respiratory Insufficiency Questionnaire (SRI) scores (*n*=69) in patients without impaired autonomy (= no impairment level)

|  | **Mean** | **SD** | **95% CI**  **Lower limit** | **Upper limit** |
| --- | --- | --- | --- | --- |
| Respiratory complaints | 57.1 | 20.1 | 52.3 | 62.0 |
| Physical Functioning | 48.1 | 20.7 | 43.1 | 53.1 |
| Attendant Symptoms and Sleep | 65.4 | 22.4 | 60.0 | 70.9 |
| Social Relationships | 74.7 | 21.6 | 69.5 | 79.9 |
| Anxiety | 55.0 | 25.3 | 48.9 | 61.1 |
| Psychological Well-being | 61.8 | 18.7 | 57.3 | 66.4 |
| Social Functioning | 58.8 | 20.3 | 53.9 | 63.8 |
| Summary Scale | 60.2 | 16.0 | 56.3 | 64.0 |

CI confidence interval; SD standard deviation

Table e3: Severe Respiratory Insufficiency Questionnaire (SRI) scores (*n*=37) in patients with significantly impaired autonomy (impairment level 2)

|  | ***Mean*** | ***SD*** | ***95% CI***  ***Lower limit*** | ***Upper limit*** |
| --- | --- | --- | --- | --- |
| Respiratory complaints | 56.0 | 16.3 | 50.4 | 61.4 |
| Physical Functioning | 27.0 | 23.4 | 19.2 | 34.8 |
| Attendant Symptoms and Sleep | 54.2 | 18.1 | 48.2 | 60.3 |
| Social Relationships | 64.5 | 19.5 | 58.0 | 71.0 |
| Anxiety | 51.5 | 19.7 | 44.9 | 58.1 |
| Psychological Well-being | 56.2 | 17.5 | 50.4 | 62.1 |
| Social Functioning | 45.4 | 20.2 | 38.7 | 52.2 |
| Summary Scale | 50.7 | 13.2 | 46.3 | 55.1 |

CI confidence interval; SD standard deviation

Table e4: Severe Respiratory Insufficiency Questionnaire (SRI) scores (*n*=18) in patients with seriously impaired autonomy (impairment level 3)

|  | **Mean** | **SD** | **95% CI**  **Lower limit** | **Upper limit** |
| --- | --- | --- | --- | --- |
| Respiratory complaints | 39.4 | 17.9 | 30.5 | 48.3 |
| Physical Functioning | 19.9 | 17.5 | 11.2 | 28.6 |
| Attendant Symptoms and Sleep | 46.8 | 21.3 | 36.2 | 65.6 |
| Social Relationships | 55.6 | 20.2 | 45.5 | 65.6 |
| Anxiety | 40.3 | 23.2 | 28.8 | 51.8 |
| Psychological Well-being | 46.5 | 21.3 | 35.9 | 57.0 |
| Social Functioning | 34.7 | 22.5 | 23.6 | 45.9 |
| Summary Scale | 40.4 | 16.4 | 32.3 | 48.6 |

CI confidence interval; SD standard deviation

Table e5: Patient characteristics and NIV data in nursing home patients (*n*=7).

| (n=7) | |
| --- | --- |
| N of males (%) | 4 (57%) |
| Age (years) | 69.0 ± 6.8 |
| Body-mass index (kg/m²) | 27.9 ± 8.2 |
| Level of autonomy impairment (N/%)   - Significant - Serious - Most serious | 3  3  1 |
| Time under NIV (years) | 1 ± 0.8 |
| NIV initiation (N/%) |  |
| - Chronic elective NIV | 2 (29%) |
| - Following acute NIV | 4 (57%) |
| - NIV following prolonged weaning | 1 (14%) |
